# The prevalence of burnout syndrome and its association with adherence to safety and practice standards among anesthetists working in Ethiopia

**DOI:** 10.1016/j.amsu.2021.102777

**Published:** 2021-09-03

**Authors:** Tikuneh Yetneberk, Meseret Firde, Dinberu Eshetie, Abebe Tiruneh, Jolene Moore

**Affiliations:** a Department of Anesthesia and Critical Care, Debre Tabor University, Debre Tabor, Ethiopia; b School of Medicine Medical Sciences and Nutrition, University of Aberdeen, Aberdeen, UK

**Keywords:** Anesthetist, Burnout, Emotional exhaustion, Ethiopia, AOR Adjusted Odds Ratio, BSc Bachelor of Science degree, COR Crude Odds Ratio, MBI-HSS Maslach Burnout Inventory, Human Service Survey, MSc Master of Science degree

## Abstract

**Background:**

Burnout amongst healthcare professionals is a serious challenge affecting health care practice and quality of care. The ongoing pandemic has highlighted this on a global level. This study aimed to determine the prevalence of burnout syndrome and its association with adherence to safety and practice standards among non-physician anesthetists in Ethiopia.

**Methods:**

A cross-sectional survey was conducted amongst non-physician anesthetists throughout Ethiopia in January 2020 utilizing an online validated questionnaire containing sociodemographic characteristics, symptoms of burnout using the 22 items of the Maslach Burnout Inventory-Human Services Survey (MBI-HSS) scale, 10 questions designed to evaluate the best practice of providers, and 7 questions evaluating self-reported errors. The MBI-HSS questions assessed depersonalization, emotional exhaustion, and personal accomplishment. A high level of burnout was defined as a respondent with an emotional exhaustion score ≥27, a depersonalization score ≥10, and a personal accomplishment score ≤33 in the MBI-HSS subscales. Bi-variable and multivariable logistic regression were used to identify factors associated with burnout.

**Results:**

Out of a total of 650 anesthetists approached, 400 responded, a response rate of 61.5%. High levels of burnout were identified in 17.3% of Ethiopian anesthesia providers. Significant burnout scores were found in academic anesthetists (*p* = 0.01), and were associated with less years of anesthesia experience (*p* < 0.001), consuming >5 alcoholic drinks per week (*p* = 0.02), and parenthood (*p* = 0.01).

**Conclusion:**

We found that non physician anesthetists working in Ethiopia is suffering by high levels of burnout. The problem is alarming in those working at academic environments and less experienced.

## Highlights

• Burnout were identified in 17.3% of Ethiopian anesthesia providers.
• Significant burnout scores were found in academic anesthetists.
• Anesthetists who have high burnout score committed medical malpractice.

## 1 Background

Burnout is a syndrome characterized by emotional exhaustion, depersonalization, and loss of sense of achievement that results from prolonged exposure to job stressors [[1], [2], [3], [4]]. Burnout in healthcare professionals is a global challenge affecting health care practice and quality of care which has reached epidemic levels [[5], [6], [7]]. Although often overlooked, burnout remains a real problem in the medical profession and can lead to reduced job satisfaction, productivity, and patient care [8].

Anesthesia is a medical profession with a high prevalence of stress due to the nature of the daily work environment [4,6,9]. Due to its position on the front line of patient care, burnout is very frequent among anesthetists [10,11]. Besides its effect on the professionals’ health, burnout has been linked with tense professional relationships in team members, together with lower work activity, worse quality of care, and higher health care costs [10,[12], [13], [14]].

Anesthetists at a high risk of burnout have reported more frequent medication errors, mistakes with negative consequences for patients, less attention to patients, a trend to leave the practice, drug addiction, and suicidal ideation [11]. In Ethiopia, anesthetists report low job satisfaction (42.5%) and a high turnover intention (47.8%) rate, signalling that anesthetists are dissatisfied with their jobs and suffer from low morale and motivation [15,16].

Physicians are highly affected by burnout; a systematic review reports the prevalence of burnout is highly varied across studies (10%–41% high risk, up to 59% at least moderate risk) [6]. A high level of burnout has been consistently associated with the strained working pattern, working as a younger consultant, and having children, but no consistent relationship has been found between burnout and hospital characteristics, gender, or marital status [6]. Burnout is a real phenomenon in anesthesia and approximately 25% of anesthetists are at high risk of developing it [9]. A high burnout score has been noted in young anesthetists, and associated with fewer years of anesthetic experience, and female gender [8]. This study aimed to determine the prevalence of burnout syndrome and its association with adherence to safety and practice standards among non-physician anesthetists in Ethiopia.

## 2 Materials and methods

A cross-sectional nationwide survey was distributed to 650 qualified anesthetists working across Ethiopia in January 2020. Anesthetists' contact details were obtained through the Ethiopian Association of Anesthetists social media networking group ‘Telegram’ channel. An invitation to participate and a link to the online survey, created using Survey Monkey software, was then sent via email. Survey completion was voluntary and confidential. To assure the confidentiality of the participants, the survey was set up to delink the responses to the respondents' IP address or account. The software created a unique identifier to prevent multiple responses from a single participant. The questionnaire was divided into 4 parts and contained 46 questions. A combination of multiple-choice questions and Likert scales quantifying respondents' level of agreement with a statement, were used.

The first section of the questionnaire was designed to capture demographic, social, and work characteristics. The second section of the questionnaire was the Maslach Burnout Inventory-Human Services Survey (MBI-HSS) [17]. The MBI-HSS includes 22 questions: 5 assessing depersonalization, 9 for emotional exhaustion, and 8 for personal accomplishment. A score is given to each part of the MBI-HSS, based on a frequency scale of 0 (‘never’) to 6 (‘every day’). A high level of burnout was defined as a respondent with an emotional exhaustion score ≥27, depersonalization score ≥10, and personal accomplishment score ≤33 in these MBI-HSS subscales [8,[18], [19], [20]].

The third section of the questionnaire included 10 questions designed to evaluate behaviors commonly identified as best practices in anesthesia, adapted from the work of previous investigators [19]. Questions were evaluated using a 5-point Likert scale (never, rarely, sometimes, often, and always). The fourth section of the questionnaire included 7 questions that evaluated the frequency of self-reported errors using questions developed by previous investigators. The frequency was evaluated using a 5-point Likert scale (never, once, sometimes, multiple times, often). The surveys were administered in English language. The questionnaire was tested for reliability and internal validity. The internal consistency estimate of the reliability of the test score (Cronbach's alpha) was 0.89, indicating a good construct of the questionnaire.

SPSS version 20 software was used for statistical analysis. In addition to simple descriptive statistics, both bivariable and multivariable logistic regression were used to identify factors associated with the burnout level of anesthetists. In the multivariable analysis, variables with a p-value <0.05 were considered statistically significant. Our work is fully compliant with the STROCSS criteria [1]. It was register with unique identifying number researchregistry7086.

Ethical approval was obtained from the academic committee of the department of anesthesia, Debre Tabor University. Written informed consent was obtained from each study participant.

## 3 Results

Out of a total of 650 anesthetists approached, 400 responded (response rate of 61.5%); of these 95% (n = 380) of participants were male and 71.5% (n = 286) were within the 20–30-year age group. Most (67.8%; n = 271) were single. The majority of the anesthetists had less than 5 years’ experience in clinical practice (64%; n = 256), reported working greater than 40 h per week (67.5%; n = 270), and were in academic roles (59.3%; n = 237). The prevalence of burnout amongst responding anesthetists in Ethiopia was 17.3% (n = 69) (Table 1).

**Table 1:** Socio-demographic characteristics and prevalence of burnout syndrome amongst study participants.

Variable	Number (%)
Age	
20–30 years	286(71.5)
31–40 years	114(28.5)
Sex	
Male	380(95)
Female	20(5)
Marital status	
Married	129(32.3)
Single	271(67.8)
Role in Academia	
Yes	237(59.3)
No	163(40.8)
Level of Education	
Bachelor of Science (BSc)	235(58.8)
Master of Science (MSc)	165(41.3)
Average working hours per week	
10–20 h	23(5.8)
21–40 h	107(26.8)
>40 h	270(67.5)
Consume >5 alcohol drinks per week	
Yes	200(50)
No	200(50)
Obtain recognition from managers	
Yes	95(23.8)
No	305(76.3)
Cigarette smoking	
Yes	15(1.8)
No	185(96.3)
Work Experience	
Less than 1 year	57(14.2)
1–5 years	199(49.8)
6–10 years	117(29.3)
Greater than 10 years	27(6.8)
Parenthood status	
Yes	176(44)
No	224(56)
Burnout	
Yes	69(17.1)
No	331(82.8)

In this survey, 8.8% (n = 35) of anesthetists reported performing procedures without appropriate training and 31.3% (n = 53) reported that they fall short in the quality of care they provide ‘multiple times. In addition, 4.3% (n = 17) agreed they do not monitor the patient in the operating room as closely as they should ‘multiple times’ or ‘often’, 4.8% (n = 19) make mistakes without negative consequences to their patients ‘multiple times’ and 43.1% (n = 172) had made a medication error at least once in the last year (Table 2).

**Table 2:** Response distribution to individual questions evaluating anesthetists self-reported errors and quality of care.

Individual items	Likert scale, Number (%)				
	Never	Once	Sometimes	Multiple times	Often
I make mistakes without negative consequences to patients	63(15.8)	92(23)	222(55.5)	19(4.8)	4(1)
I perform procedures without appropriate training	220(55)	31(7.8)	114(28.5)	35(8.8)	0(0)
I make mistakes with negative consequences to patients	278(69.5)	117(29.3)	5(1.3)	0(0)	0(0)
I fall short in the quality of care I provide to my patients	117(29.3)	23(5.8)	207(51.7)	53(31.3)	0(0)
I do not have enough time or attention to my patients	207(51.7)	47(11.8)	132(33)	4(1)	10(2.5)
I do not monitor the patient in the operating room as closely as I should	279(69.8)	10(2.5)	94(23.5)	13(3.3)	4(1)
I have made medication errors involving the wrong drug or dose in the last year	228(57)	167(41.8)	5(1.3)	0(0)	0(0)

Fewer years of work experience, parenthood, alcohol, and academic roles were found to be significantly associated with a high level of burnout in the multivariate analysis (Table 3). Anesthetists with less than 5 years of work experience were more likely to experience a high level of burnout compared to those with more than or equal to 5 years of experience (AOR: 27.87, 95%CI: 6.21–125.04). Anesthetists in an academic role were 4.38 times more likely to experience a high level of burnout compared to their non-academic counterparts.

**Table 3:** Bivariate and multiple logistic regression analysis of Maslach Burnout Inventory scores among anesthetists.

Variables	Burnout			
	**COR**	**p-value**	**AOR**	**p-value**
Educational level				
BSc	1.76 (1.01–3.07)	0.047	3.22 (0.96–10.73)	0.057
MSc	1		1	
Experience(years)				
< 5	2.54 (1.36–4.76)	0.040	27.87 (6.21–125.04)	<0.001
≥5	1		1	
Parenthood status				
Yes	2.29 (1.35–1.89)	0.002	3.28 (1.22–8.84)	0.019
No	1		1	
Average drinks per week				
≥ 5	2.21 (0.73–6.76)	0.163	5.23 (1.29–21.19)	0.021
< 5	1		1	
Academic role				
Yes	1.52(0.9–2.56)	0.115	4.38 (1.42–13.49)	0.01
No	1		1	

COR=Crude Odds Ratio, AOR = Adjusted Odds Ratio, 1 = constant.

Anesthetists who reported consuming on average greater than or equal to 5 alcoholic drinks per week were more likely to experience a high level of burnout compared to those consuming on average less than 5 alcoholic drinks per week (AOR: 5.23, 95%CI: 1.29–21.19). In addition, anesthetists who were parents were 3.28 times more likely to experience a high level of burnout. Educational level (higher or lower) was not significantly associated with a high level of burnout (P = 0.057).

Anesthetists exhibiting a high level of burnout reported more frequent mistakes with negative consequences to patients, performing procedures without appropriate training, and less attention to patients compared with anesthetists with no burnout syndromes (Table 4).

**Table 4:** Anesthetists self-reported errors and quality of care by the presence of burnout.

Questions	Burnout	Distribution of response, Number (% of a row)				
		Never	Once	Sometimes	Multiple times	Often
I make mistakes without negative consequences to patients	Yes	0	14(20.3)	51(73.9)	4(5.8)	0
	No	63(19)	78(23.6)	171(51.7)	15(4.5)	4(1.2)
I perform procedures without appropriate training	Yes	18(26.1)	4(5.8)	32(46.4)	15(21.7)	0
	No	202(61)	27(8.2)	82(24.8)	20(6)	0
I make mistakes with negative consequences to patients	Yes	36(52.5)	33(47.8)	0	0	0
	No	242(73.1)	84(25.4)	5(1.5)	0	0
I fall short in the quality of care I provide to my patients	Yes	10(14.5)	5(7.2)	54(78.3)	0	0
	No	107(32.3)	18(5.4)	153(46.2)	53(16)	0
I do not have enough time or attention to my patients	Yes	5(7.2)	0	46(66.7)	18(26.1)	0
	No	202(61)	4(1.2)	86(26)	29(8.8)	0
I do not monitor the patient in the operating room as closely as I should	Yes	60(87)	4(5.8)	5(7.2)	0	0
	No	219(66.2)	9(2.7)	89(26.9)	10(3)	4(1.2)
I have made medication errors involving the wrong drug or dose in the last years	Yes	18(26.1)	51(73.9)	0	0	0
	No	210(63.4)	116(35)	5(1.5)	0	0

In this survey, anesthetists exhibiting a high level of burnout reported low adherence with best anesthesia practice standards. Of those who met the criteria for burnout syndrome, 4.3% n = 3) never performed a complete machine check prior to commencing anesthesia or double-checked medications and were less likely to ‘always’ perform best practice actions (Table 5).

**Table 5:** Performance of best practice of anesthesia by the presence of burnout.

Questions	Burnout	Distribution of response, Number (% of a row)				
		Never	Rarely	Sometimes	Often	Always
Do you visit patients preoperatively?	Yes	0	0	34(49.3)	13(18.8)	22(31.9)
	No	5(1.5)	51(15.4)	61(18.4)	39(11.8)	175(52.9)
Do you check the results of preoperative investigations?	Yes	0	0	0	42(60.9)	27(39.1)
	No	8(2.4)	0	43(13)	43(13)	237(71.6)
Do you read about the next day's surgery and the patient's disease?	Yes	0	34(49.3)	20(29)	5(7.2)	10(14.5)
	No	14(4.2)	43(13)	115(34.7)	66(19.9)	93(28)
Do you perform a complete machine check at the beginning of the day?	Yes	3(4.3)	4(5.8)	34(49.3)	5(7.2)	23(33.3)
	No	5(1.5)	26(7.9)	78(23.6)	54(16.3)	168(50.8)
Do you double-check medication vials for correct administration?	Yes	3(4.3)	9(13)	25(36.2)	0	32(46.4)
	No	0	37(11.2)	55(16.6)	48(14.5)	191(57.7)
Do you make sure the monitor alarms are enabled before the administration of anesthetics?	Yes	0	34(49.3)	12(17.4)	4(5.8)	19(27.5)
	No	10(3)	63(19)	91(27.5)	73(22.1)	94(28.4)
Do you Confirm that surgery will be performed on the correct side?	Yes	0	5(7.2)	37(53.6)	0	27(39.1)
	No	0	13(3.9)	113(34.1)	52(15.7)	153(46.2)
Do you wear a gown, gloves, and mask for spinal/epidurals?	Yes	0	25(36.2)	9(13)	9(13)	26(37.7)
	No	51(15.4)	70(21.1)	33(10)	36(10.9)	141(42.6)
Do you review the postoperative conditions of your patient?	Yes	0	27(39.1)	23(33.3)	9(13)	10(14.5)
	No	0	96(29)	120(36.3)	66(19.9)	49(14.8)

## 4 Discussion

This study investigated the prevalence of burnout syndrome and its association with adherence to safety and practice standards among anesthetists working in Ethiopia. The prevalence of this condition in different healthcare fields has been evaluated yet there remains an absence of information on burnout amongst Ethiopian anesthetists which we sought to address. Our findings reveal that 17.3% of participating anesthetists have a high level of burnout.

The level of burnout in this study was found to be lower than other studies in different groups of health professionals using the MBI-HSS scale which report prevalence ranging from 21% to 57% [[2], [3], [4], [5]], yet was higher than findings from a Polish study conducted among anesthesia providers with burnout in 12.06% [6], and a Brazil study of anesthesiologists with rates of 11.2% [2]. The differences may be attributed to, or influenced by, the differing career pathways and nature of the health-care ecosystems.

Previous studies have identified demographic variables such as experience, alcohol drinking habit, parenthood status, and academic roles as some of the predictive factors for a high level of burnout [3,5]. In line with these, our study found that having <5 years of work experience, consuming on average greater than or equal to 5 alcoholic drinks per week, working in an academic environment, and having children were all associated with burnout symptoms.

In contrast, some studies identified female sex [3], high workload [3], and young professionals age between 30 and 39 ^2^ as predictive factors for a high level of burnout. Anesthetists at risk of burnout are reported as having committed errors frequently and adhered to safety standards poorly [3,7]. Anesthetists at high risk of burnout also report more frequent medication errors [8]. In correlation with previous studies, our study found self-perceived medical errors and poor adherence with anesthesia practice standards were common in anesthetists who developed burnout syndromes. A high prevalence of burnout among health care professionals is of concern as it appears to affect quality, safety, and performance.

The strength of this study is the first in its kind by addressing the burnout syndrome in Ethiopian non-physician anesthetist and this study is limited by the constraints of self-reported surveys including accuracy dependent on thorough and reliable completion. Through utilizing the Ethiopian Association of Anesthetists’ common networking group, we were able to obtain a large sample size.

Considerable expansion of surgical services has taken place in Ethiopia over recent years, and despite capacity building initiatives there continues to be a shortage of anesthesia providers nationwide. The wellbeing of this limited anesthetic workforce is paramount to the delivery of safe anesthesia.

## 5 Conclusions

We found that non physician anesthetists working in Ethiopia is suffering by high levels of burnout. The problem is alarming in those working at academic environments and less experienced.

## Ethical approval

Ethical approval was taken from Debre Tabor university.

## Sources of funding

No source of funding.

## Author contribution

**Tikuneh Yetneberk**: participate in study concept or design, data collection, data analysis or interpretation, writing the paper. **Meseret Firde**: participate in study concept or design and data collection. **Denberu Eshetie**: participate in study concept or design, data collection and data analysis. **Abebe Tiruneh**: participate in study concept or design, data collection and interpretation. **Jolene Moore**: participate in study concept or design, data collection and writing the paper.

## Registration of research studies

1. Name of the registry:

Unique Identifying number or registration ID: researchregistry7086.

2. Hyperlink to your specific registration (must be publicly accessible and will be checked):

## Guarantor

Tikuneh Yetneberk.

## Consent for publication

Not applicable.

## Competing interests

The authors declare no competing interests.

## Funding

None obtained.

## Availability of data and material

All the data were presented in this article.

## Provenance and peer review

Not commissioned, externally peer-reviewed.

## Declaration of competing interest

None.
